# Indicators of active disease and steroid dependency in patients with inflammatory bowel diseases not treated with biologics in a German real-world-setting

**DOI:** 10.1007/s00384-020-03588-w

**Published:** 2020-05-18

**Authors:** B. Bokemeyer, M. Ghiani, A. Fuchs, B. Deiters, F. Hardtstock, A. Brandes, J. Knop, H. D. Orzechowski, T. Wilke

**Affiliations:** 1 Gastroenterology Practice Minden, Minden, Germany; 2 IPAM e.V, Wismar, Germany; 3 AOK PLUS, Dresden, Germany; 4 GWQ ServicePlus AG, Düsseldorf, Germany; 5 Takeda Pharma Vertrieb GmbH & Co. KG, Berlin, Germany; 6 Ingress-Health HWM GmbH, Alter Holzhafen 19, 23966 Wismar, Germany

**Keywords:** Inflammatory bowel disease (IBD), Non-biologic treatment, Immunosuppressant treatment, Steroids, Ulcerative colitis (UC), Crohn’s disease (CD)

## Abstract

**Background and aims:**

While a minority of inflammatory bowel disease (IBD) patients receives biologics in Germany, little is known about therapeutic needs of patients receiving non-biologic therapies. This study aimed to identify indicators of active disease/steroid dependency in patients with moderate to severe Crohn’s disease (CD) and ulcerative colitis (UC) treated with conventional therapies and to describe health care resource use (HCRU)/cost.

**Methods:**

CD/UC patients treated with immunosuppressants (IS) and/or systemic or locally acting oral corticosteroids (CS) were identified in German claims data (2013–2017) and followed for 12 months post-therapy start. Indicators of active disease/steroid dependency during follow-up period were (i) ≥ 2 prescriptions of CS (sensitivity ≥ 4) or (ii) ≥ 1 IBD-related surgery or (iii) > 7 days IBD-related hospitalization(s).

**Results:**

Of 9871 included IBD patients (5170 CD, 4701 UC), 25.7%/19.9% (CD/UC) received ≥ 2 prescriptions of CS (sensitivity, 17.4%/15.7%) (i), 3.2% experienced IBD-related surgeries (ii), and 2.5% > 7 days of hospitalizations (iii). Altogether, 44.4% had indicators of active disease/steroid dependency (sensitivity, 23.9%). Among patients with active disease/steroid dependency, 78.0% received CS monotherapy at baseline. Of these, 89.6% received a CS monotherapy in the follow-up period, too. Proportionally, fewer patients with CS monotherapy (57.4%) than IS therapy (91.0%) visited a specialist. HCRU/cost per patient year was significantly higher in patients with than without active disease/steroid dependency.

**Conclusions:**

A substantial percentage of biologic-naïve IBD patients suffers from active disease/steroid dependency. The majority receives a monotherapy with systemic CS. Referral to gastroenterologists for treatment optimization is recommended, also because active disease/steroid dependency is associated with increased HCRU/cost.

**Electronic supplementary material:**

The online version of this article (10.1007/s00384-020-03588-w) contains supplementary material, which is available to authorized users.

## Introduction

Crohn’s disease (CD) and ulcerative colitis (UC) are the most common forms of inflammatory bowel diseases (IBD) and can be characterized by chronic intestinal inflammation with mucosal lesions. The prevalence of IBD is estimated to surpass 0.4% in western countries, with substantial burden for modern health care systems [1, 2]. In Germany, it is estimated that more than 320,000 people suffer from IBD [3]. Health-related quality of life (HRQoL) of IBD patients is substantially affected by the physical strain and psychosocial impairments due to these diseases [4].

The primary IBD treatment goal is long-lasting corticosteroid (CS)-free disease remission with a good HRQoL [5, 7]. To reach this objective, therapy with non-biologic and biologic immunosuppressants (IS) is indicated in case of moderate to severe CD and UC [6–9]. In case of failure of first-line treatment, second-line therapies should be considered. The decision regarding which of the available agents to prescribe should be based on the course of the disease as well as existing disease activity, primary or secondary non-response, antibody formation, side effects with first-line therapy, comorbidities, and patient preferences [6–8, 10, 11].

Several previous studies addressed the real-world treatment of moderate-to-severe IBD patients receiving biologic therapies [12–16]. However, in Germany as in most other western European countries, the majority of IBD patients are treated with conventional non-biologic therapies [17]*.* Treatment with non-biologic drugs has not been studied in detail over the recent years. That is why our study aimed to describe the treatment of IBD patients with at least moderate disease severity in Germany treated with conventional therapies only, but not with biologics. Specifically, in these patients, we assessed whether there were still indicators of active disease and/or steroid dependency as observed by specific drug prescriptions and/or IBD-related surgeries or hospitalizations. Assessment of indicators of active disease/steroid dependency in this patient population is important from a clinical point of view as any observed active disease/steroid dependency might implicate a need for a therapy change or intensification. Furthermore, active disease/steroid dependency might be associated with increased health care resource use (HCRU) and cost. Therefore, we additionally compared HCRU and cost between these biologic-naïve patients with and without indicators for active disease/steroid dependency.

## Methods

We conducted a retrospective claims data analysis using data provided by several statutory health insurance funds geographically distributed in Germany (AOK PLUS and insurance funds represented by service provider GWQ ServicePlus AG). As these data include diagnoses and services across all health care sectors with no study-related selection bias, they are therefore representative of the German health care context. The database included about 8.5 million insured lives, which reflects 11.8% of the German population insured by statutory health insurance funds.

The dataset provided information on patient demographics (age, gender, date of death), outpatient treatment (diagnosis codes, procedures, and visits to GPs and specialists), inpatient treatment (admission and discharge dates, diagnosis codes, procedures, length of stay), and claims filed for prescription medications. Clinical information, such as instruments to assess the severity of disease or disease activity (e.g., Crohn’s Disease Activity Index, Mayo Score, Harvey Bradshaw Index, and laboratory values) was not available in the dataset. Our data covered four consecutive years from 1 July 2013 to 30 June 2017 (GWQ data, due to data availability: until 31 December 2016). All continuously insured patients with an age of at least 18 years and a confirmed diagnosis of CD or UC (ICD-10 K50.-, K51.-; M2Q-criterion: at least one inpatient or two outpatient diagnoses in two different quarters of the study period) were included. Patients with one inpatient or two outpatient diagnoses of both CD and UC were excluded as well as patients with a double diagnosis of CD or UC and indeterminate colitis (ICD-10 K52.3).

As patients with moderate to severe IBD disease severity were addressed, we included patients who received at least one prescription of a non-biologic IS (azathioprine (ATC L04AX01), mercaptopurine (ATC L01BB02), methotrexate (ATC L01BA01 and L04AX03)) and/or a systemic corticosteroid (ATC H02) or oral budesonide (ATC A07EA06, excluding non-oral applications such as foams, based on application form information derived from the respective central pharmaceutical numbers, see Supplemental Table 2) in the index period between 1 July 2015 to 30 June 2016 (GWQ data, due to data availability: until 31 December 2015). In consequence, patients with mild disease who did not require treatment with IS or systemic corticosteroids (CS) have been excluded. Further, all patients who received a biologic during the index or follow-up period were excluded, as treatment with biologics was already analyzed in a recent study based on the same dataset [17]. Patients with prescriptions of calcineurin inhibitors were also excluded as this is a last-line treatment after biologics in most cases.

**Fig. 1:**
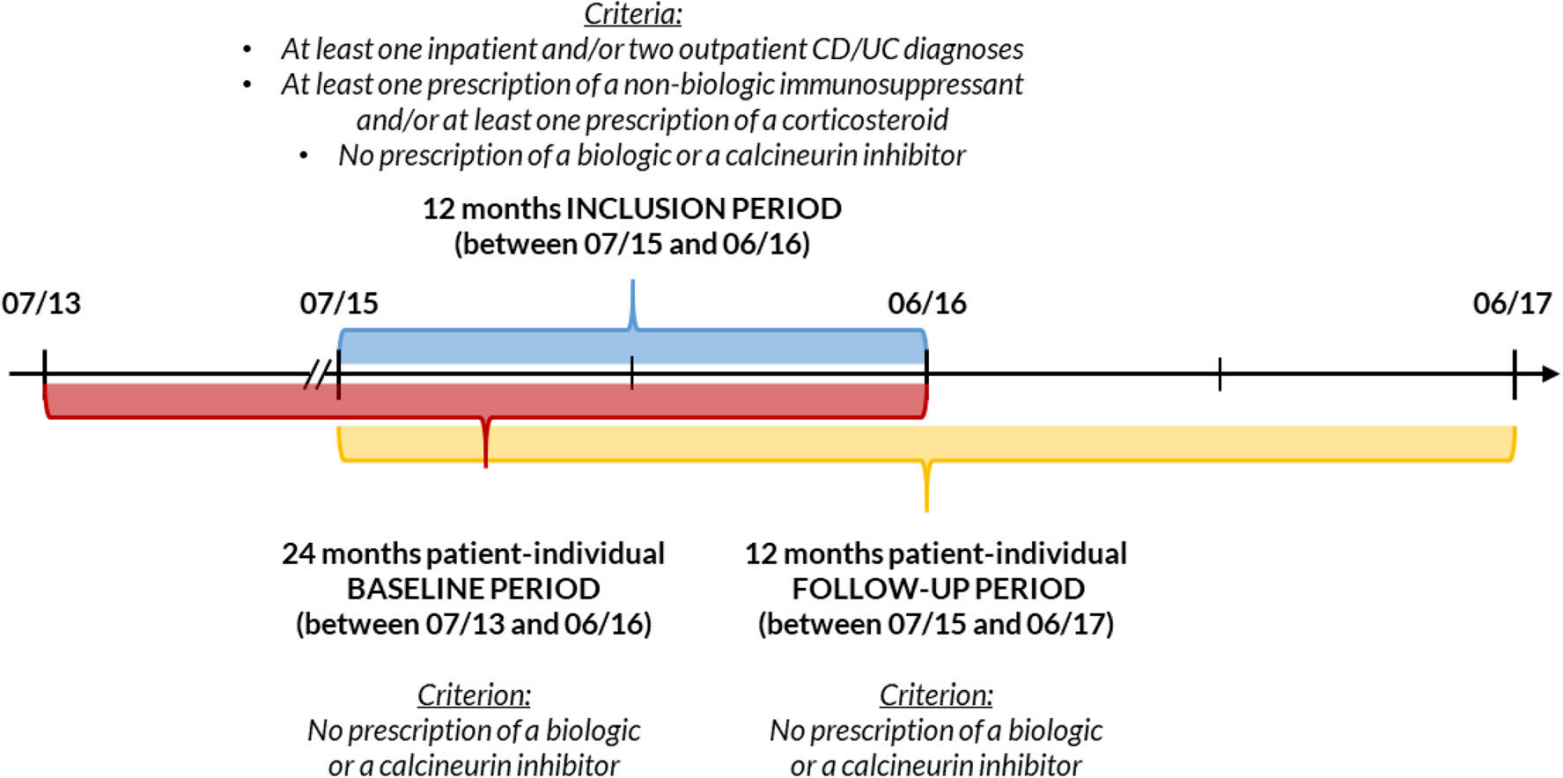
Study methodology. The timeframe of patient-individual baseline period (24 months) and follow-up period (12 months) in this study. Patients were considered for inclusion between July 2015 and June 2016. To illustrate, for a patient with the first prescription of a non-biologic immunosuppressant on 1 June 2016 (index date), baseline characteristics were observed between 1 June 2014–31 May 2016 (24 months period). HCRU and cost as well as indicators of active disease/steroid dependency were measured in the 12-months following the index date (2 June 2016–1 June 2017). *CD*, Crohn’s disease; *UC*, ulcerative colitis

Observation started with the date of the first prescription of index non-biologic IS or CS during the inclusion period and lasted 12 months (or until death if the patient died within the follow-up period; Fig. 1). Baseline characteristics were reported either at the index date (age, gender) or were reported for a 24-month baseline period (for IBD-related complications and other disease and treatment characteristics see Supplemental Table 1). Baseline characteristics and treatments were not used to assess active disease/steroid dependency.

### Definition of active disease/steroid dependency

Active IBD disease and/or steroid dependency was assumed to exist if at least one of the following criteria during the 12 months follow-up period was met:

- ≥ 2 prescriptions of systemic CS (ATC: H02AB-/H02B; sensitivity, ≥ 3/≥ 4 prescriptions),
- ≥ 2 prescriptions of locally-acting oral budesonide (ATC: A07EA-, only drug codes corresponding to oral use were used; sensitivity, ≥ 3/≥ 4 prescriptions),
- at least one inpatient stay for IBD-related surgery (operation and procedure (OPS) codes 5-45 – incision, excision, resection and anastomosis of small intestine/colon, 5-46 – other surgery of the small intestine/colon, 5-48 – surgery of the rectum, 5-49 – surgery of the anus),
- IBD-related hospitalizations (ICD10: K50.-/K51.-) of more than 7 days in total in the follow-up period.

### Healthcare resource utilization and cost

We reported CS drug therapy including CS dosages in prednisolone equivalents, IBD-related hospitalizations (with IBD as the main diagnosis or IBD-associated complications, see Supplemental Table 1), visits to general practitioners (GP) and specialists, as well as sick leave days associated with an IBD diagnosis.

Both all-cause and IBD-associated direct and indirect costs (direct: drug cost, outpatient, and inpatient treatment cost; indirect: cost due to sick leave days) were calculated. Direct cost calculation was based on list prices for drugs as documented in the database (pharmacy sales price at date of filling a prescription), outpatient costs of services based on coded physicians’ uniform rating scale (EBM) numbers, and hospitalizations based on DRG-based reimbursement. Indirect costs associated with sick leave days were estimated based on official statistics on the average gross real salaries in Germany by gender, age, and year [18]. As the most recent available data by gender and age were from 2014, observed growth rates in the overall salaries were used to project the growth of salaries within gender and age subgroups to the year 2017. Average gross salaries per day were multiplied by the length of the leave to derive the overall cost.

### Statistical analysis

All reported variables for patient characteristics, treatment and dosage patterns, HCRU, and costs were analyzed using descriptive statistics, including mean, median, ranges, and standard deviation. Patient characteristics as well as HCRU and cost were compared between CD and UC patients with and without indicators for active disease/steroid dependency by means of suitable parametric and non-parametric tests (*t* test, chi-square, Mann-Whitney *U*) and, additionally, based on a multivariate logistic regression model. To account for early death of some patients, drug dosages, HCRU, and cost were reported per observed patient year. Analyses were performed using SAS (Version 9.4), SPSS (Version 24), STATA (Version 14.1), and MySQL (Version 8.0).

Because the present study was non-interventional, had a retrospective design, and was based on anonymized data, informed consent of patients was not required. This is in accordance with German law and policies of the institutions assessing patient-level data (IPAM, GWQ ServicePlus, and AOK PLUS). The study was evaluated by a scientific steering committee to which all the authors belonged and was based on a study protocol approved before start of data analysis.

## Results

### Patient selection

**Fig. 2:**
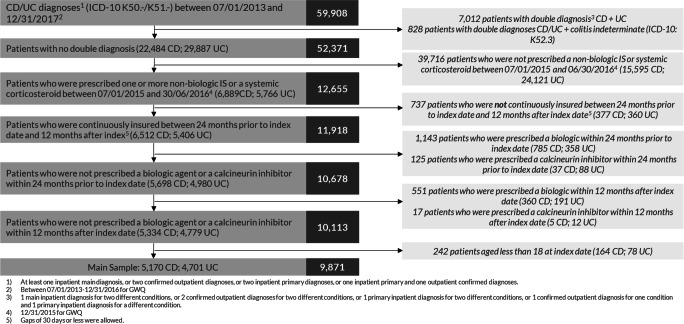
Study population attrition chart. Based on defined inclusion/exclusion criteria, Fig. 2 shows the selection of patient samples. *CD*, Crohn’s disease; *IS*, immunosuppressants; *UC*, ulcerative colitis

Among 59,908 identified IBD patients in the database, 39,716 patients (CD 15,595; UC 24,121) were excluded due to the fact that they did not receive any non-biologic IS or systemic CS in the inclusion period (Fig. 2). Frequently prescribed medications in this group were antibacterial agents for systemic use (ATC: J01; 4476 CD patients and 6842 UC patients), aminosalicylates (ATC: A07EC; 2131 CD patients and 7525 UC patients), oral corticosteroids (ATC: A07EA; 55 CD patients and 666 UC patients), and agents acting on the renin-angiotensin system (ATC: C09; 3372 CD patients and 7453 UC patients). Additionally, excluded patients due to pre-defined exclusion criteria such as the use of biologics are shown in Fig. 2.

Mean age of the included 5170 CD patients was 48.2 years, and that of the 4701 UC patients 55.0 years; 56.9% and 48.5% were female, respectively (Table 1). 41.8% of CD and 30.7% of UC-patients received at least one prescription of a non-biologic IS in the baseline period (in combination with or without CS), whereas 83.4% and 87.3%, respectively, received CS (systemic CS or oral budesonide).

**Table 1:** Patients’ baseline characteristics

	All patients		All CD patients		CD patients with active disease/steroid dependency		CD patients w/o active disease/steroid dependency		*p* value*	All UC patients		UC patients with active disease/steroid dependency		UC patients w/o active disease/steroid dependency		*p* value*
*N*	9871		5170		2443		2727			4701		1940		2761		
Age in years, mean (SD)	51.5	(17.1)	48.2	(16.0)	50.3	(16.3)	46.4	(15.5)	< 0.001	55.0	(17.5)	58.2	(17.5)	52.8	(17.2)	< 0.001
Female, gender, *N* (%)	5220	(52.9)	2940	(56.9)	1350	(55.3)	1590	(58.3)	0.027	2280	(48.5)	942	(48.6)	1338	(48.5)	0.948
Pensioner status, *N* (%)																
Pensioner	2878	(29.2)	1227	(23.7)	693	(28.4)	534	(19.6)	< 0.001	1651	(35.1)	867	(44.7)	784	(28.4)	< 0.001
Non-pensioner	6993	(70.8)	3943	(76.3)	1750	(71.6)	2193	(80.4)	< 0.001	3050	(65.9)	1073	(55.3)	1977	(71.6)	< 0.001
CCI, mean (SD)	1.8	(2.5)	1.5	(2.3)	1.8	(2.5)	1.2	(2.0)	< 0.001	2.2	(2.8)	2.7	(3.0)	1.8	(2.5)	< 0.001
IBD-related medications, *N*^1^ (%)																
Immunosuppressants	3605	(36.5)	2163	(41.8)	624	(25.5)	1539	(56.4)	< 0.001	1442	(30.7)	341	(17.6)	1101	(39.9)	< 0.001
Steroids	8418	(85.3)	4314	(83.4)	2374	(97.2)	1940	(71.1)	< 0.001	4104	(87.3)	1906	(98.2)	2198	(79.6)	< 0.001
IBD-related surgery, *N* (%)	590	(6.0)	406	(7.9)	192	(7.9)	214	(7.9)	0.988	184	(3.9)	104	(5.4)	80	(2.9)	< 0.001
IBD-related hospitalization, *N* (%)	1983	(20.0)	1234	(23.9)	705	(28.9)	529	(19.4)	< 0.001	749	(15.9)	387	(20.0)	362	(13.1)	< 0.001

Descriptive patient characteristics of the overall sample as well as of the UC and CD sample with and without indicators of active disease/steroid dependency. All characteristics refer to either index date (age, gender) or the 24 months baseline period. *CCI*, Charlson Comorbidity Index; *CD*, Crohn’s disease; *UC*, ulcerative colitis. ^1^ At least one prescription in the baseline period. *Differences between patients with and without evidence for active disease/steroid dependency were assessed by chi-square test for categorical variables or by Mann-Whitney *U*/*t* test for continuous variables

### Indicators of active disease/steroid dependency in the follow-up period

Among the included 5170 CD patients, 21.8% received at least two prescriptions of systemic CS, 26.9% received at least two prescriptions of oral budesonide, 4.1% experienced at least one IBD-associated surgery, and 3.0% spent more than 7 days in total in hospital during the 12 months follow-up period. Taking double counting into account, this led to an overall number of 47.3% of observed CD patients with indicators of active disease/steroid dependency (Table 2).

**Table 2:** Number and percentage of UC and CD patients with indicators of active disease/steroid dependency

	Overall sample		CD patients		UC patients	
*N*	9871		5170		4701	
≥ 2 prescriptions of systemic CS in the follow-up, *N* (%)	2539	(25.7)	1127	(21.8)	1412	(30.0)
≥ 2 prescriptions of oral budesonide in the follow-up, *N* (%)	1964	(19.9)	1390	(26.9)	574	(12.2)
IBD-related inpatient surgery in the follow-up, *N* (%)	315	(3.2)	213	(4.1)	102	(2.2)
IBD-related hospitalization(s) > 7 days in the follow-up, *N* (%)	244	(2.5)	157	(3.0)	87	(1.9)
Patients with evidence for active disease/steroid dependency (any of the above), *N* (%)	4383	(44.4)	2443	(47.3)	1940	(41.3)

Describes the total number and percentage of patients with evidence for active disease/steroid dependency, in the overall sample, as well as in the CD and UC samples. Note: the follow-up period corresponds to the 12 months after index date. As index date, the first prescription of a CS or a non-biologic IS (whatever came first) in 1 July 2015–30 June 2016 was used. *CD*, Crohn’s disease; *CS*, corticosteroids; *IBD*, inflammatory bowel disease; *UC*, ulcerative colitis

In a sensitivity analysis with at least four systemic CS and/or oral budesonide prescriptions within the 12 months follow-up as the threshold to identify active disease/steroid dependency, 27.3% of CD patients showed indicators of active disease/steroid dependency. The difference to the base case is driven by lower patient numbers with ≥ 4 systemic CS prescriptions (8.1%) and ≥ 4 oral budesonide prescriptions (16.1%) (Suppl. Table 4; additional sensitivity analysis for ≥ 3 prescriptions in Suppl. Table 3).

In comparison to above CD patients, among the observed 4701 UC patients, more patients (30.0%) received at least two prescriptions of systemic CS, whereas less patients received at least two prescriptions of oral budesonide (12.2%), experienced at least one IBD-associated surgery (2.2%), and spent more than 7 days in total in hospital (1.9%). Overall, 41.3% of UC patients had an indicator of active disease/steroid dependency (Table 2). In the sensitivity analysis, 20.1% of UC patients showed indicators of active disease/steroid dependency (12.2% with ≥ 4 systemic CS prescriptions, 5.6% with ≥ 4 oral budesonide prescriptions) (additional sensitivity analysis for ≥ 3 prescriptions in Suppl. Table 4).

### Comparison of patient characteristics with and without indicators of active disease/steroid dependency

Patients with indicators of active disease/steroid dependency were older, had a greater number of comorbidities, received more often CS monotherapy (as compared to non-biologic IS), and experienced more IBD-associated surgeries and/or hospitalizations in the baseline period compared to patients without indicators of active disease/steroid dependency (Table 1). This was confirmed in multivariable logistic regression analyses (Table 3).

In CD patients, the following baseline characteristics were associated with a higher risk for indicators of active disease/steroid dependency in the follow-up period: age of at least 60 years, male gender, higher rate of comorbidities (Charlson Comorbidity Index (CCI)), no prescription of conventional IS in the baseline period, at least one CS prescription in the baseline period, and at least one CD-related hospitalization as well as at least one hospitalization due to IBD-related complications. An inpatient IBD-related surgery in the baseline period (incision, excision, resection and anastomosis of small intestine/colon, other surgery of the small intestine/colon, surgery of the rectum, and surgery of the anus) was associated with a lower risk of being classified as having active disease/steroid dependency in the CD patient sample.

Factors found to be significantly associated with the risk to be classified as having active disease/steroid dependency in UC were the same as for CD patients, with exception of IBD-related surgery and hospitalizations due to IBD-related complications at baseline as well as gender (Table 3).

### Drug treatment patterns of patients with indicators of active disease/steroid dependency

**Fig. 3:**
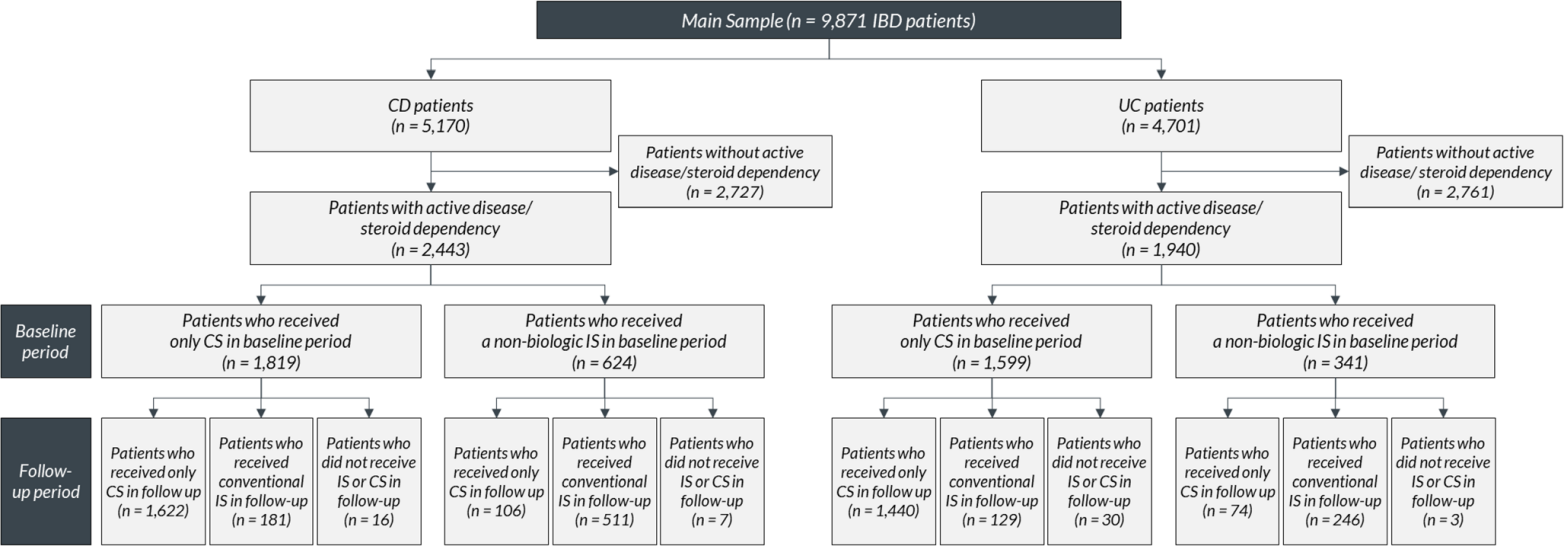
Drug treatment patterns of patients with indicators of active disease/steroid dependency. Fig. 3 reports, among CD and UC patients with indicators of active disease/steroid dependency, drug treatment patterns both in the baseline (24 months before index date), and follow-up period (12 months after index). Specifically, treatment with either systemic CS/oral budesonide or non-biologic IS is shown. Other treatments such as biologics (patients who received biologics were excluded from the initial analysis sample), 5-ASA or locally acting non-oral CS are not shown. *CD*, Crohn’s disease; *CS*, corticosteroids; *IBD*, inflammatory bowel disease; *IS*, immunosuppressants; *UC*, ulcerative colitis

Baseline and follow-up treatments of patients with indicators of active disease/steroid dependency are shown in Fig. 3. Among the 2443 CD patients with indicators of active disease/steroid dependency, 1819 patients (74.5%) received a systemic CS and/or oral budesonide and no IS in the 24 months baseline period (including index date), whereas 624 patients (25.5%) received at least one prescription of an IS. Among patients with CS/oral budesonide monotherapy, the majority also received a CS/oral budesonide monotherapy in the follow-up period (1622 patients, i.e., 66.4% of all CD patients with indicators of active disease/steroid dependency). We observed a lower percentage of patients visiting a gastroenterological specialist at least once in the CS/oral budesonide monotherapy cohorts: 63.8% of CD patients with indicators of active disease/steroid dependency who received systemic CS and/or oral budesonide monotherapy in the study period visited a specialist in the observed 12 months, compared to 93.4% in CD patients with indicators of active disease/steroid dependency who received an IS.

Among 1940 UC patients with indicators of active disease/steroid dependency, 1599 patients (82.4%) received a systemic CS/oral budesonide and no non-biologic IS in the 24 months baseline period (including index date), whereas 341 patients (17.6%) received at least one prescription of an IS (Fig. 3). Among patients with CS/oral budesonide monotherapy, the majority received a CS/oral budesonide monotherapy in the follow-up period as well (1440 patients, i.e., 74.2% of all UC patients with indicators of active disease/steroid dependency), whereas 129 (6.6%) received an IS and 30 patients (1.5%) neither received a systemic CS/oral budesonide nor an IS. Again, percentage of patients who visited a specialist at least once was lower in patients with CS/oral budesonide monotherapy in both the baseline and follow-up periods, compared to those who received CS/oral budesonide in the baseline period but an IS in the follow-up period (51.9% versus 87.6%).

During the observational period, the 2443 CD patients with indicators of active disease/steroid dependency (Fig. 3) received on average 7.3 mg prednisolone equivalents of systemic CS per observed patient day, and 10.9% of patients received > 10 mg. The 1940 UC patients with indicators of active disease/steroid dependency received on average 7.4 mg prednisolone equivalents of systemic CS per observed patient day and 17.8% of patients received > 10 mg.

### Healthcare resource utilization and cost

Table 4 outlines HCRU associated with the treatment of CD and UC patients in the follow-up period and compares it between those with and without indicators of active disease/steroid dependency. Based on CD patients with indicators of active disease/steroid dependency, 29.8% experienced at least one hospitalization during the observed 12 months with a mean number of 0.6 CD-associated hospitalizations per patient year; 13.7% of CD patients without indicators of active disease/steroid dependency were hospitalized during that period (0.2 hospitalizations per patient year). In addition to that, outpatient GP and specialist visit frequencies as well as sick leave days were higher in the patient group with indicators of active disease/steroid dependency (Table 4).

**Table 3:** Logistic regression model estimating factors associated with the probability to observe indicators of active disease/steroid dependency in a patient (active disease/steroid dependency as defined in the main scenario (see Table 1))

Baseline characteristics	Model based on all patients (*N* = 9871)		Model based on CD patients (*N* = 5170)		Model based on UC patients (*N* = 4701)	
	Pseudo-*R*^2^ = 0.1561		Pseudo-*R*^2^ = 0.1717		Pseudo-*R*^2^ = 0.1376	
	*N*	OR (95% CI)	*N*	OR	*N*	OR
Age at index ≥ 60 years	3069	1.341 (1.204–1.494)	1223	1.231 (1.051–1.442)	1846	1.454 (1.256–1.684)
Male gender	4651	1.179 (1.081–1.286)	2230	1.268 (1.122–1. 433)	2421	1.111 (0.976–1.264)
IBD Type = CD	5170	1.577 (1.430–1.739)	–	–	–	–
CCI	9871	1.050 (1.030–1.071)	5170	1.063 (1.030–1.097)	4701	1.039 (1.012–1.066)
No prescription of conventional IS in the baseline period	6266	1.843 (1.659–2.047)	3007	2.080 (1.812–2.388)	3259	1.531 (1.303–1.798)
At least one prescription of CS in the baseline period	8418	8.162 (6.547–10.174)	4314	7.820 (5.958–10.263)	4104	8.832 (6.068–12.857)
IBD-related surgeries	590	0.748 (0.613–0.912)	406	0.557 (0.433–0.717)	184	1.230 (0.890–1.702)
At least one hospitalization with K50 as main diagnosis in baseline period	1116	1.588 (1.366–1.846)	1116	1.684 (1.440–1.970)	0	–
At least one hospitalization with K51 as main diagnosis in baseline period	722	1.484 (1.250–1.761)	0	–	722	1.425 (1.201–1.690)
At least one hospitalization due to IBD-related complications in the baseline period	219	2.575 (1.838–3.608)	181	3.245 (2.202–4.781)	38	1.752 (0.841–3.651)

Shows the outcome of logistic regression models to estimate the probability to observe active disease/steroid dependency in all IBD patients and separately for CD and UC patients. All baseline characteristics refer to either index date (age, gender) or the 24 months baseline period. *CCI*, Charlson Comorbidity Index; *CD*, Crohn’s disease; *CS*, corticosteroids; *IBD*, inflammatory bowel disease; *IS*, immunosuppressants; *GP*, general practitioner; *OR*, odds ratio; *UC*, ulcerative colitis

HCRU differences between UC patients with and without indicators of active disease/steroid dependency were similar: 19.8% of UC patients with indicators had at least one UC-related hospitalization (0.3 hospitalizations per patient year) whereas this was 10.9% (0.2 hospitalizations per year) in those without indicators. Outpatient visit and sick leave day differences were also similar to the CD patient sample.

**Fig. 4:**
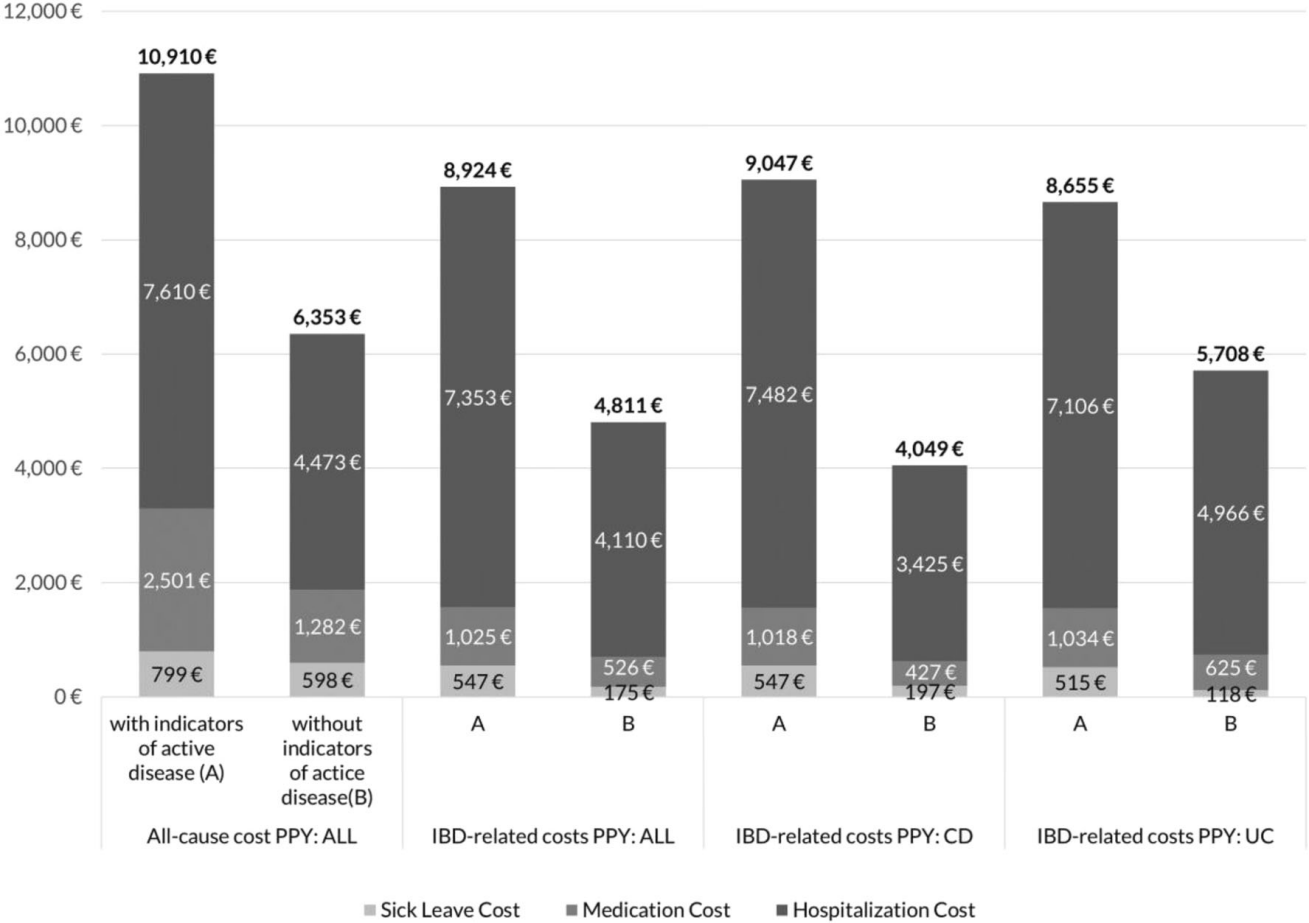
All-cause and IBD-associated cost of treatment of IBD patients with and without active disease/steroid dependency. Figure 4 reports all-cause direct and indirect cost for all patients (CD + UC). Furthermore, both direct and indirect cost associated with IBD treatment is reported separately for all as well as CD and UC patients. Specifically, IBD drug cost, inpatient treatment cost, as well as cost associated with IBD sick leave days are reported. Sick leave cost calculation is based on official statistics regarding daily salary. Comparisons are done between patients with/without indicators of disease activity/steroid dependency, based on the base scenario as presented in this manuscript. *CD*, Crohn’s disease; *IBD*, inflammatory bowel disease; *PPY*, per patient year; *UC*, ulcerative colitis

Based on all patients, all-cause and IBD-associated cost of IBD patients with indicators of active disease/steroid dependency (10,910€ and 8924€, respectively) were higher than those for patients without active disease (6353€ and 4811€). Among CD patients, the difference in CD-associated cost between patients with indicators for active disease/steroid dependency and patients without such indicators was 9047€ versus 4049€ (*p* < 0.001), respectively, with CD-hospitalization cost as the most important cost driver (7482€ versus 3425€, *p* < 0.001). Similarly, among UC patients, the difference in UC-associated cost between the two patient groups was 8655€ versus 5708 € (*p* < 0.001), with UC hospitalization cost again as the most important cost driver (7106€ versus 4966€, *p* < 0.001; Fig. 4).

## Discussion

The main objective of this study was to assess active disease and/or steroid dependency in moderate to severe IBD patients who were not treated with biologics. We indirectly assessed active disease and steroid dependency based on coded treatment in a 12-month follow-up period. The study compared treatment patterns, HCRU, and cost between patients with and without indicators of active disease/steroid dependency.

It is assumed that treatment of IBD patients as well as associated HCRU and cost are strongly influenced by the quality of care. Specialized centers typically participate in prospective observational studies or registries, whereas less-specialized practices are underrepresented in former studies of specialized IBD-centers. For the purposes of this study, it was important to assess outcomes of IBD patients unaffected by study site or selection bias and to include treatments prescribed by non-specialists such as GPs. Therefore, we decided to analyze a representative claims data set provided by statutory health insurance funds. The main strength of this database, in addition to absence of any selection bias, is that all outpatient prescriptions are covered. Secondly, services are included across all health care sectors. And thirdly, the data set is complete regarding treatments, HCRU, and cost, allowing generalizability of our results.

A total of 39,716 of the initially identified patients (CD: 15,595; UC: 24,121) were finally excluded from our main sample since they did not receive any biologic or non-biologic IS or systemic CS in the inclusion period. Frequently prescribed medications in this group were antibacterial agents for systemic use, aminosalicylates, and locally acting CS for topical use. Due to these treatment patterns, we interpret above excluded patients to be patients who were responsive to 5-ASA and/or suffered from mild disease only. In addition, we excluded patients who received a biologic either in the baseline or follow-up period. The main reason was that this study aimed to address non-biologic treatments only. Data about effectiveness and safety of biologic treatments as well as persistence and switch rates have already been explored in an earlier study based on the same dataset [17]. In this study, 1248 patients (1020 anti-TNFα; 228 VDZ) were included, 837 of them were bio-naïve (773 anti-TNFα/64 VDZ). A substantial percentage of these patients received a higher biologic dosage in the maintenance phase than recommended in the summary of product characteristics. Still, 30–40% received a CS therapy and/or experienced at least one IBD-associated hospitalization in a year.

Three main conclusions can be drawn from our analysis. First, in our cohort of IBD patients with at least moderate disease, we observed a substantial percentage of patients with indicators of remaining active IBD disease and/or steroid dependency. Second, over a period of at least 3 years, most of these patients received a CS monotherapy, which deviates from guideline recommendations [6, 7]. Third, active disease/steroid dependency as defined in this study was associated with a substantial increase in HCRU and cost compared to patients without indicators.

The assessment of active disease/steroid dependency was, due to the nature of the study, based on hospital and prescription data. However, as we chose conservative thresholds, we believe that our approach identifies IBD active disease and steroid dependency with good validity. The majority of the patients with indicators of active disease/steroid dependency was assigned to this group because of CS use. Based on guidelines and clinical practice, at least two systemic CS prescriptions and/or two prescriptions of oral budesonide were interpreted as a strong signal for IBD active disease/steroid dependency. Average daily doses of 7.3 mg (in CD) and 7.4 mg (in UC) over a period of 12 months and the fact that 10.9% of CD and 17.8% of UC patients received a dosage of at least 10 mg prednisolone equivalents per day [19] are a strong signal of a relevant CS-use in this group, and is far away from the therapeutic goal of a steroid-free remission as required in the guidelines [6, 7]. To address a potential remaining uncertainty, we ran sensitivity analyses. By applying a strict assumption of a minimum of four instead of two systemic CS prescriptions and/or four instead of two prescriptions of oral budesonide, still one-fifth of UC patients and one-fourth of CD patients showed indicators of active disease/steroid dependency.

Within the group of IBD patients with indicators for active disease/steroid dependency, we observed a substantial percentage receiving a CS monotherapy over a period of 3 years. 66.4% of CD patients and 74.2% of UC patients with indicators of disease activity received a CS therapy in the 24 months baseline and 12 months follow-up period only, i.e., for three consecutive years. Long-term CS use is associated with serious risks, including osteoporosis, cardiovascular disease, alterations in glucose, and psychiatric disturbances [20, 21]. Patients receiving CS monotherapy are not treated according to guidelines which postulate the treatment objective of CS-free long-term treatment and generally state that CS are not indicated for maintenance therapy due to the high risks and therapeutic ineffectiveness in the maintenance period [6–9].

Treatment of patients with indicators of active disease/steroid dependency was associated with a substantially higher HCRU and cost in our study, including indirect cost due to IBD-associated sick days. We conclude from these numbers that a revision of the treatment of these patients might increase medication cost due to added non-biologic IS or biologics, but has the potential to lead to HCRU and/or cost savings especially around hospitalization and sick days cost.

Due to the lack of recent publications addressing a similar IBD population, we were not able to compare our findings to previous literature. Older publications analyzed real-world effectiveness and safety of non-biologic treatments in CD and UC patients [22–25], but, to our knowledge, a cohort-based multi-center assessment of the non-biologic real-world treatment of IBD patients with at least moderate disease severity has not been done so far.

Despite the strengths of our study, we also acknowledge limitations. First, we were able to assess the non-biologic treatment history of our patients for a baseline period of 24 months only. If patients received a non-biologic IS or biologic earlier than that, they were wrongly assigned to the CS monotherapy group. Second, clinical information concerning disease severity and activity as well as laboratory values were not available. Proxy codes were used to account for this. Also, documentation on the reasons why physicians prescribed specific treatments and dosages of IS/CS were not available. Third, our conclusions about the prescribed CS dosage were based on prescription patterns only. Finally, we might have over-estimated the percentage of patients receiving a CS monotherapy as we required at least one prescription of CS or a non-biologic IS as inclusion criterion, to address patients with at least moderate IBD severity. So, as CS use was one of the inclusion criteria and, at the same time, an indicator of active disease/steroid dependency, this might have led to a pre-selection of patients with active disease. On the other hand, active disease/steroid dependency was only assumed if a patient received ≥ 2 (sensitivity analysis ≥ 3 or ≥ 4) CS prescriptions.

**Table 4:** HCRU of CD and UC patients with and without indicators of active disease/steroid dependency

	All patients (*N* = 9871)			CD patients (*N* = 5170)			UC patients (*N* = 4701)		
	With active disease/steroid dependency	W/o active disease/steroid dependency	*p* value^a^	With active disease/steroid dependency	W/o active disease/steroid dependency	*p* value^a^	With active disease/steroid dependency	W/o active disease/steroid dependency	*p* value^a^
*N*	4383	5488		2443	2727		1940	2761	
At least one IBD-related hospitalization, %	25.4	12.3	< 0.001	29.8	13.7	< 0.001	19.8	10.9	< 0.001
IBD-related hospitalizations rate PPY	0.5	0.2	< 0.001	0.6	0.2	< 0.001	0.3	0.2	< 0.001
IBD-related surgeries, %	0.06	–	–	0.08	–	–	0.04	–	–
At least one IBD-related outpatient visit, %									
GP	86.9	85.6	0.055	90.1	88.3	0.034	82.9	82.9	0.987
Specialist	64.6	63.5	0.229	70.0	67.9	0.107	57.9	59.1	0.422
IBD-related outpatient visits rate PPY									
GP	6.4	5.9	< 0.001	6.8	6.1	< 0.001	5.9	5.7	0.080
Specialist	7.3	6.8	< 0.001	7.7	6.9	< 0.001	6.7	6.6	0.379
IBD-related sick leave days PPY	0.2	0.1	< 0.001	0.2	0.1	< 0.001	0.2	0.1	0.010

Shows the HCRU in the overall sample as well as in the CD and UC samples, separately for patients with and without indicators of active disease/steroid dependency. All characteristics refer to either index date (age, gender) or the 24 months baseline period. *CD*, Crohn’s disease; *IBD*, inflammatory bowel disease; *GP*, general practitioner; *PPY*, per patient year; *UC*, ulcerative colitis; *w/o*, without. ^*a*^*p* values refer to tests of differences between patients with evidence for active disease/steroid dependency versus patients with no such evidence. Differences in proportions were tested with a chi-squared test. Differences in event rates were tested with an exact probability test

## Conclusions

According to our data, in clinical practice, a substantial percentage of at least 24 to 44% (depending on thresholds used for CS use) of moderate to severe IBD patients without a biologic therapy show indicators of active disease. Most of these patients (66.4% of CD and 74.2% of UC patients) receive a therapy with systemic CS and/or oral budesonide without immunosuppressants over a period of at least 3 years. Referral of these patients to gastroenterologists or specialized IBD-centers is strongly recommended to optimize treatment, also because active disease is associated with substantial HCRU and cost increases.

## Electronic supplementary material

ESM 1 (DOCX 13 kb)

ESM 2 (DOCX 16 kb)

ESM 3 (DOCX 14 kb)

ESM 4 (DOCX 14 kb)

## Data Availability

Due to German data protection law (SGB X), we are not allowed to distribute the analyzed dataset.
