# Supplementary material for: Indicators of active disease and steroid dependency in patients with inflammatory bowel diseases not treated with biologics in a German real-world-setting
Source: Int J Colorectal Dis. 2020 May 18;35(8):1587–98. doi: 10.1007/s00384-020-03588-w (PMC7340655; doi:10.1007/s00384-020-03588-w)
Supplement: Supplementary file 1 — (DOCX 13 kb) [file 384_2020_3588_MOESM1_ESM.docx]

Supplemental Table 1: ICD-10 codes used to identify IBD-related complications / extraintestinal manifestations

| **ICD-10** | **Complications and extraintestinal manifestations** |
| --- | --- |
| K60.- | Fistulas and fissures |
| K61.- | Abscesses |
| H20.0 | Acute and subacute Iridocyclitis |
| K83.0 | Cholangitis |
| L52.- | Erythema nodosum |
| L88.- | Pyoderma gangraenosum |
