# Supplementary material for: Indicators of active disease and steroid dependency in patients with inflammatory bowel diseases not treated with biologics in a German real-world-setting
Source: Int J Colorectal Dis. 2020 May 18;35(8):1587–98. doi: 10.1007/s00384-020-03588-w (PMC7340655; doi:10.1007/s00384-020-03588-w)
Supplement: Supplementary file 2 — (DOCX 16 kb) [file 384_2020_3588_MOESM2_ESM.docx]

Supplemental Table 2: ATC-codes for IBD-related non-biologic medication

| **Medication** | **ATC-code** |
| --- | --- |
| *Corticosteroids* | |
| Corticosteroids for systemic use | H02- |
| Oral budesonide | A07EA06-  Please note: To reflect oral application forms, drug prescriptions within the range H02 have only been included, if the dosage form acronym that was derived from the Central Pharmaceutical Number indicated an oral route of administration. This includes:   - tablets (RET, SMT) - capsules (KAPS, KMP, KMR, HVW, HKM, REK, REKA, MRKA) - granulates (GRA, GMR) |
| *Non-biologic immunosuppressants* | |
| Azathioprine | L04AX01 |
| Mercaptopurine | L01BB02 |
| Methotrexate | L01BA01/ L04AX03 |
