# Supplementary material for: Indicators of active disease and steroid dependency in patients with inflammatory bowel diseases not treated with biologics in a German real-world-setting
Source: Int J Colorectal Dis. 2020 May 18;35(8):1587–98. doi: 10.1007/s00384-020-03588-w (PMC7340655; doi:10.1007/s00384-020-03588-w)
Supplement: Supplementary file 3 — (DOCX 14 kb) [file 384_2020_3588_MOESM3_ESM.docx]

Supplemental Table 3: Sensitivity analysis (≥3 prescriptions) - number and percentage of UC and CD patients with indicators of disease activity during follow-up

|  | **Overall sample** | | **CD patients** | | **UC patients** | |
| --- | --- | --- | --- | --- | --- | --- |
| N | 9,871 | | 5,170 | | 4,701 | |
| ≥3 prescriptions of systemic corticosteroids in the follow-up – N (%) | 1,719 | (17.4) | 740 | (14.3) | 979 | (20.8) |
| ≥3 prescriptions of oral budesonide in the follow-up – N (%) | 1,547 | (15.7) | 1,130 | (21.9) | 417 | (8.9) |
| IBD-related inpatient surgery in the follow-up – N (%) | 315 | (3.2) | 213 | (4.1) | 102 | (2.2) |
| IBD-related hospitalization(s) > 7 days in the follow-up – N (%) | 244 | (2.5) | 157 | (3.0) | 87 | (1.9) |
| **Patients with disease activity (any of the above)** – N (%) | **3,374** | **(34.2)** | **1,938** | **(37.5)** | **1,436** | **(30.6)** |
