# Supplementary material for: Indicators of active disease and steroid dependency in patients with inflammatory bowel diseases not treated with biologics in a German real-world-setting
Source: Int J Colorectal Dis. 2020 May 18;35(8):1587–98. doi: 10.1007/s00384-020-03588-w (PMC7340655; doi:10.1007/s00384-020-03588-w)
Supplement: Supplementary file 4 — (DOCX 14 kb) [file 384_2020_3588_MOESM4_ESM.docx]

Supplemental Table 4: Sensitivity analysis (≥4 prescriptions) - number and percentage of UC and CD patients with indicators of disease activity during follow-up

|  | **Overall sample** | | **CD patients** | | **UC patients** | |
| --- | --- | --- | --- | --- | --- | --- |
| N | 9,871 | | 5,170 | | 4,701 | |
| ≥4 prescriptions of systemic corticosteroids in the follow-up – N (%) | 994 | (10.1) | 419 | (8.1) | 575 | (12.2) |
| ≥4 prescriptions of oral budesonide in the follow‑up – N (%) | 1,097 | (11.1) | 833 | (16.1) | 264 | (5.6) |
| IBD-related inpatient surgery in the follow-up – N (%) | 315 | (3.2) | 213 | (4.1) | 102 | (2.2) |
| IBD-related hospitalization(s) > 7 days in the follow-up – N (%) | 244 | (2.5) | 157 | (3.0) | 87 | (1.9) |
| **Patients with disease activity (any of the above)** – N (%) | **2,354** | **(23.9)** | **1,409** | **(27.3)** | **945** | **(20.1)** |
